# Plasticity in expression of the glutamate transporters GLT-1 and GLAST in spinal dorsal horn glial cells following partial sciatic nerve ligation

**DOI:** 10.1186/1744-8069-5-15

**Published:** 2009-03-26

**Authors:** Wen-Jun Xin, Han-Rong Weng, Patrick M Dougherty

**Affiliations:** 1 Department of Anesthesiology and Pain Medicine, Division of Anesthesiology and Critical Care Medicine, University of Texas M. D. Anderson Cancer Center, 1400 Holcombe Boulevard, Unit 409, Houston, TX, 77030, USA; 2 Department of Physiology and Pain Research Center, Zhongshan Medical School, Sun Yet-Sen University, Guangzhou, 510080, PR China

## Abstract

**Background:**

Clearance of synaptically released glutamate, and hence termination of glutamatergic neurotransmission, is carried out by glutamate transporters, most especially glutamate transporter-1 (GLT-1) and the glutamate-aspartate transporter (GLAST) that are located in astrocytes. It is becoming increasingly well appreciated that changes in the function and expression of GLT-1 and GLAST occur under different physiological and pathological conditions. Here we investigated the plasticity in expression of GLT-1 and GLAST in the spinal dorsal horn using immunohistochemistry following partial sciatic nerve ligation (PSNL) in rats.

**Results:**

Animals were confirmed to develop hypersensitivity to mechanical stimulation by 7 days following PSNL. Baseline expression of GLT-1 and GLAST in naive animals was only observed in astrocytes and not in either microglia or neurons. Microglia and astrocytes showed evidence of reactivity to the nerve injury when assessed at 7 and 14 days following PSNL evidenced by increased expression of OX-42 and GFAP, respectively. In contrast, the total level of GLT-1 and GLAST protein decreased at both 7 and 14 days after PSNL. Importantly, the cellular location of GLT-1 and GLAST was also altered in response to nerve injury. Whereas activated astrocytes showed a marked decrease in expression of GLT-1 and GLAST, activated microglia showed *de novo *expression of GLT-1 and GLAST at 7 days after PSNL and this was maintained through day 14. Neurons showed no expression of GLT-1 or GLAST at any time point.

**Conclusion:**

These results indicate that the expression of glutamate transporters in astrocytes and microglia are differentially regulated following nerve injury.

## Background

Glutamate is the major excitatory neurotransmitter in the mammalian central nervous system (CNS), including the spinal dorsal horn[1]. It has been implicated in the generation and maintenance of hypersensitivity after tissue inflammation and injury [2]. Under normal physiological conditions, glutamate is released from the presynaptic membrane, and acts on glutamate receptors at the postsynaptic membrane, including N-methyl-D-aspartate (NMDA), a-amino-3-hydroxy-5-methyl-4-isoxazolepropionic acid (AMPA), and metabotropic glutamate receptors, resulting in cation influx and depolarization of the postsynaptic membrane [3]. Excessive glutamate results in an overload of Ca^2+ ^influx that can result in excitotoxicity and ultimately death of neurons. Inhibitory interneurons have been suggested as particularly vulnerable to excitotoxic damage [4].

The concentration of extracellular glutamate is tightly regulated by a family of high affinity Na^+^-dependent glutamate transporters in the cytoplasmic membrane of glial cells and to lesser extent in neurons [5,2,6]. A total of five glutamate transporters have been cloned and characterized. Among these, GLT-1 and GLAST are the major glutamate transporters in the CNS, and are mainly expressed in astrocytes [5,7-9]. Astrocytes metabolize the sequestered glutamate to glutamine using the enzyme glutamine synthetase and shuttle the newly synthesized glutamine back into neurons where it can be reconverted to glutamate [10].

Accumulating data indicate that dysfunction in glutamate transport produces marked changes in spinal processing of nociceptive inputs. Inhibition of glutamate transporters causes an elevation in spinal extracellular glutamate concentrations and produces spontaneous nociceptive behaviors and hypersensitivity to mechanical and thermal stimuli [11,12]. Deficiency and down-regulation of GLT-1 or GLAST in the spinal dorsal horn has been associated with the development of neuropathic pain induced by peripheral nerve injury [13,14] or chemotherapy [15]. It remains unclear however, whether transporter expression and function are differentially regulated in different spinal cell types. This issue was explored in this study by examination of changes in the expression and cellular localization of GLT-1 and GLAST in the spinal dorsal horn over time following partial sciatic nerve ligation (PNSL).

## Results

### Behavioral Findings

Consistent with previous results [16,17], the paw ipsilateral to the PSNL developed significant mechanical hypersensitivity as compared to the control side and to control rats. The paw withdrawal thresholds on the ipsilateral side were significantly decreased at 7 (4.6 ± 0.96 g) and 14 days (4.3 ± 1.12 g) after PSNL, compared with the control group (12.6 ± 3.84 g).

### PSNL induces activation of astrocytes in the spinal dorsal horn

**Figure 1:**
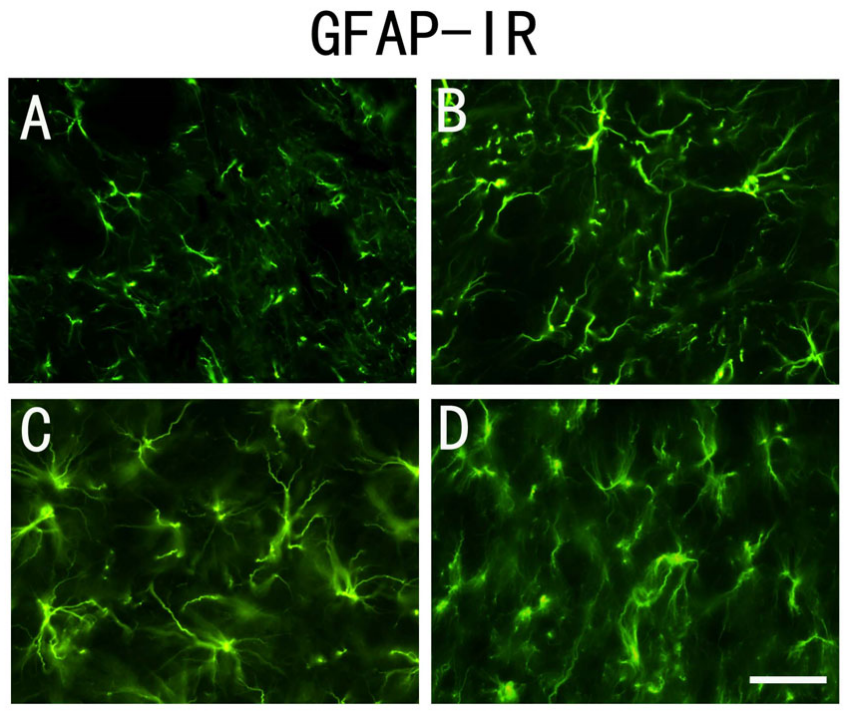
**GFAP-immunoreactivity was increased after PSNL**. A-D: GFAP-immunoreactive staining was significantly increased in spinal dorsal horn 1 day (B), 7 days (C) and 14 days (D) after PNSL compared to the control group (A). Scale bar: (A-D) = 20 μm.

Glial fibrillary acidic protein (GFAP) immunoreactivity (IR) in the dorsal horn ipsilateral to the nerve injury showed a noticeable increase by 1 day after PSNL (Figure 1B) compared with the control group (Figure 1A). This became more pronounced by day 7 and 14 after injury (Figure 1C, Figure 1D).

### GLT-1 and GLAST expression decreases in spinal astrocytes following PSNL

**Figure 2:**
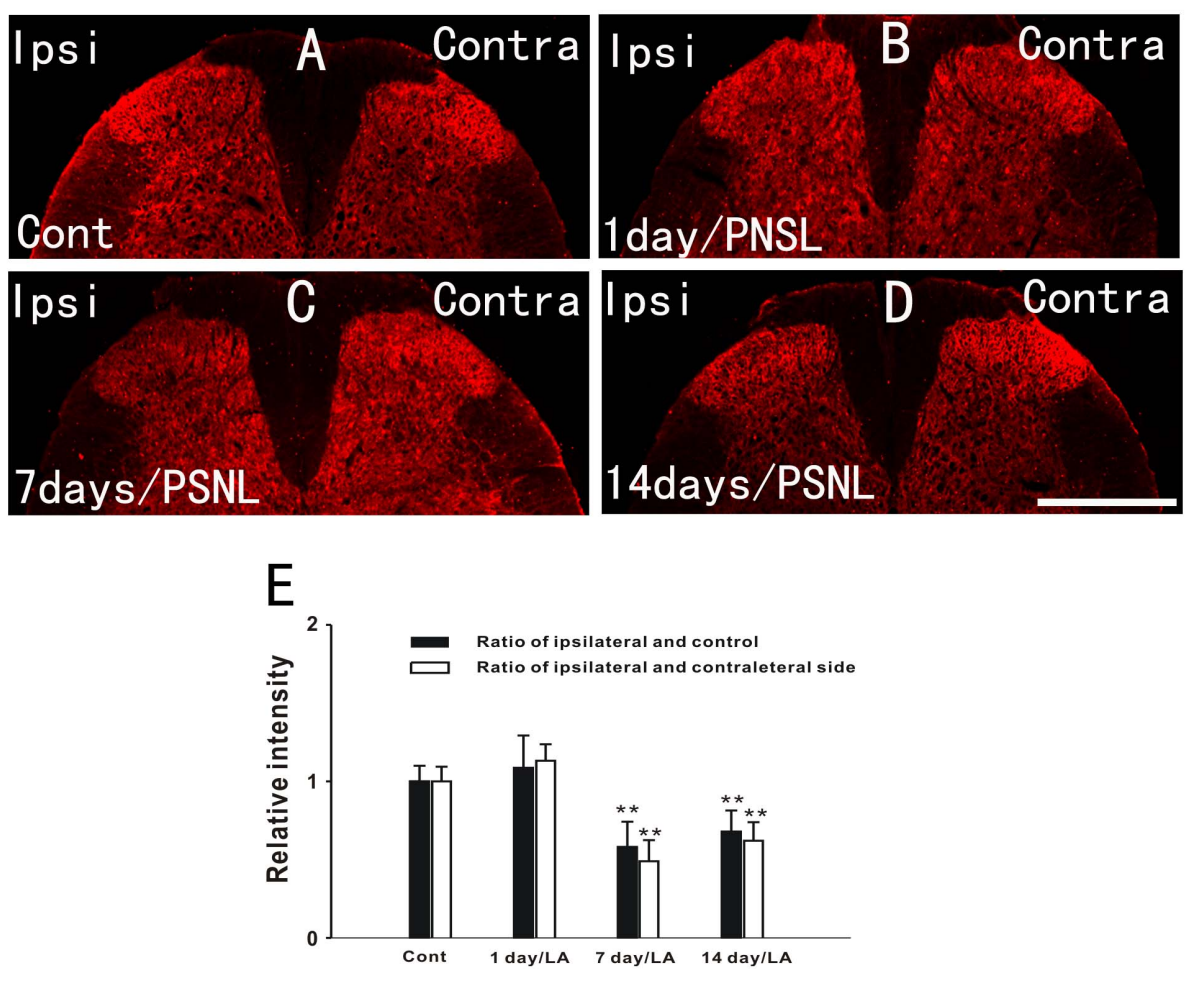
**The expression of GLT-1 was reduced after PSNL**. A-D: The results of immunohistochemmistry show that GLT-1 immunoreactivity decreased in the ipsilateral dorsal horn 7 days (C) and 14 days (D) after PNSL compared to the control group (A), However, the total amount of GLT-1 1 day after ligation did not change (B). E: Ratio (black bars) of the relative intensity between the ipsilateral sides of the variable groups (1 day, 7 days, and 14 days) and the control group as well as ratio (white bars) of the relative intensity between the ipsilateral and contralateral sides of the same variable groups (n = 5/group). Scale bar: (A-D) = 500 μm.

**Figure 3:**
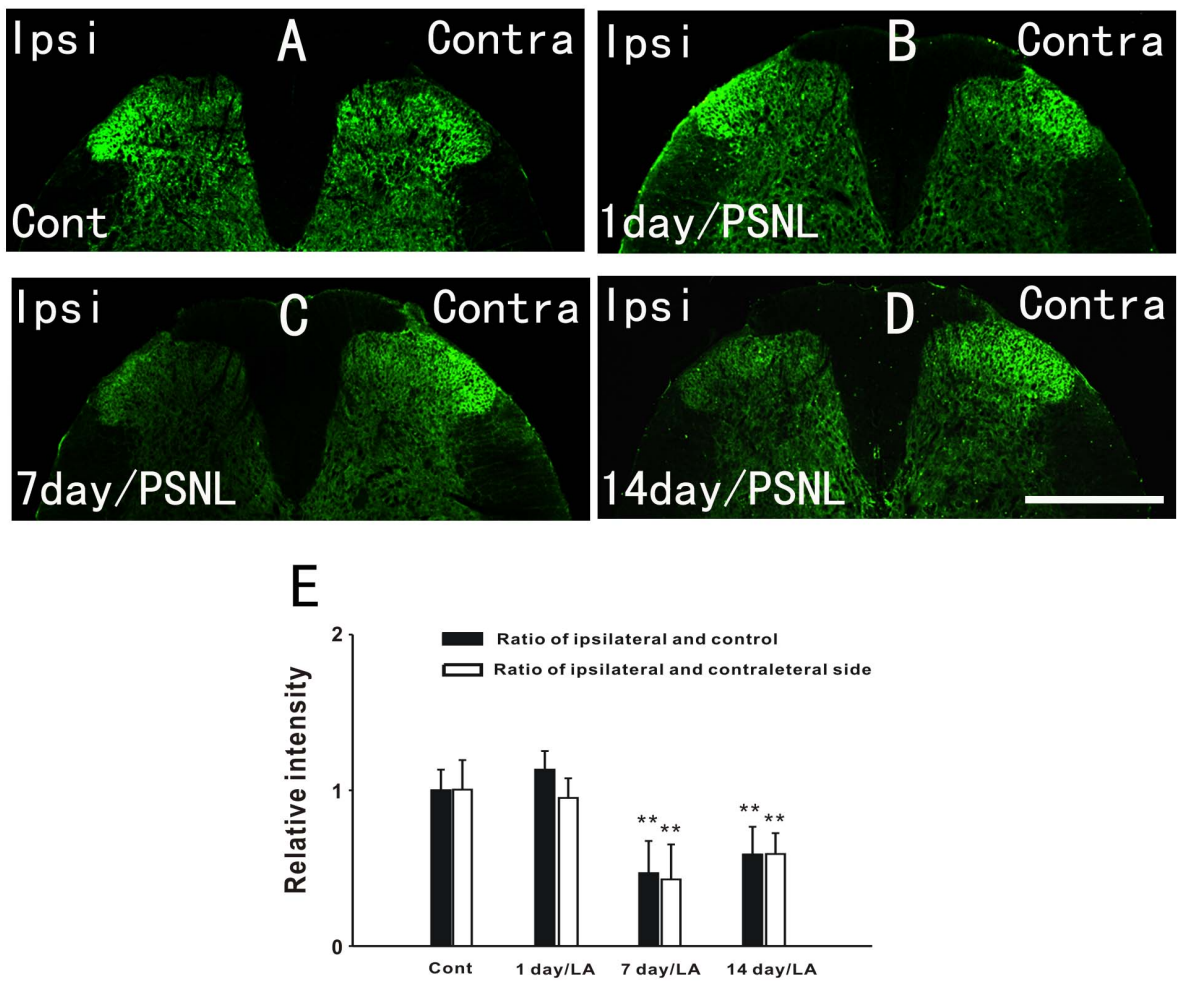
**The expression of GLAST was decreased after PSNL**. A-D: Immunohistochemistry analysis reveals that GLAST was reduced in the ipsilateral dorsal horn 7 days (C) and 14 days (D) after PNSL compared to the normal group (A), However, no change was seen in the total amount of GLAST 1 day after ligation (B). E: Ratio (black bars) of the relative intensity between the ipsilateral sides of the variable groups (1 day, 7 days, and 14 days) and the control group as well as ratio (white bars) of the relative intensity between the ipsilateral and contralateral sides of the same variable groups (n = 5/group). Scale bar: (A-D) = 500 μm.

**Figure 4:**
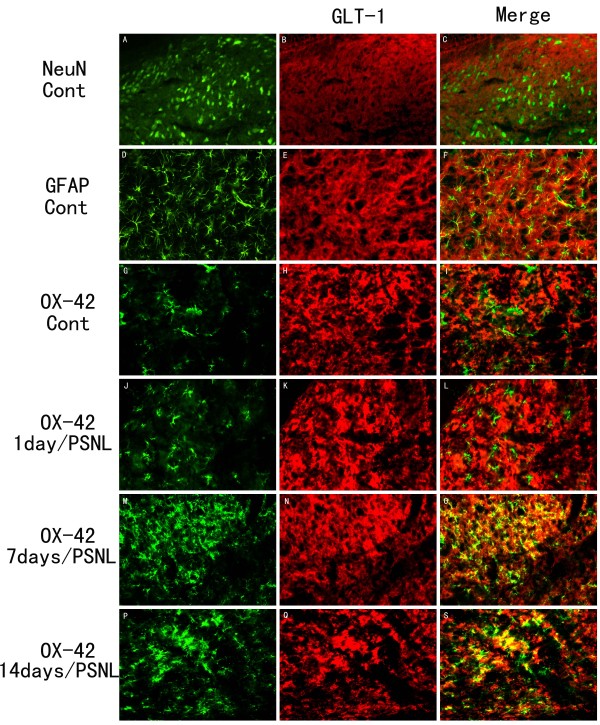
**Activated microglia expressed GLT-1 after PNSL**. A-I: Under normal conditions, the expression of GLT-1 was found in astrocytes (D-F), but not neurons (A-C) or microglia (G-I). After PSNL, microglia displayed an apparent increase in both amount and size when compared to the normal group (G, J, M, P). M-S: Microglia showed co-localization with GLT-1 antibody-positive cell in 7 (M-O) and 14 days (P-S) after PSNL, but not at 1 day after PSNL (J-L). Scale bar: (A-S) = 50 μm.

**Figure 5:**
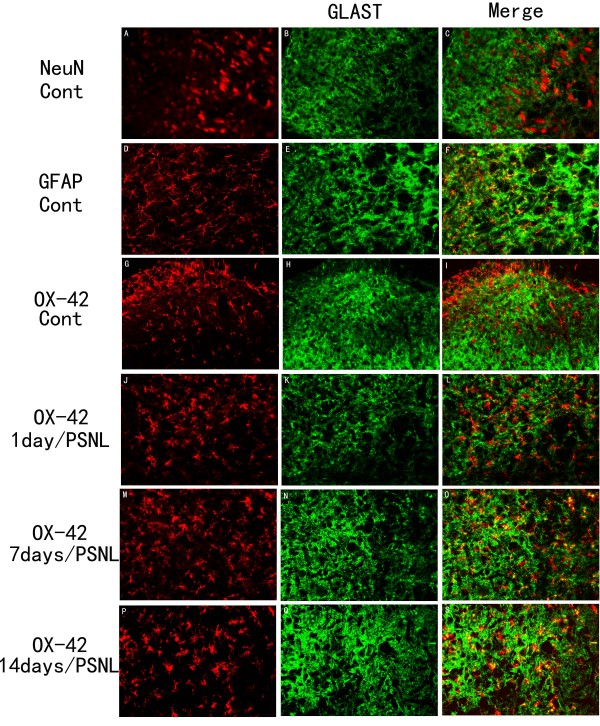
**OX-42 positive cells expressed the GLAST after PNSL**. A-I: Under normal conditions, the expression of GLAST was found in astrocytes (D-F), but not neurons (A-C) or microglia (G-I). After PSNL, microglia displayed an increase in both amount and size when compared to the normal group (G, J, M, P). M-S: The microglia was co-localized with GLAST antibody-positive cell in 7 (M-O) and 14 days (P-S) after PSNL, but not at 1 day after PSNL (J-L). Scale bar: (A-S) = 50 μm.

The expression of GLT-1 and GLAST in the spinal dorsal horn in naïve animals were mainly expressed in the gray matter of the spinal cord, especially in the superficial laminae (Figure 2A, Figure 3A). Double staining results showed that both GLT-1 and GLAST were colocalized with the astrocytic marker GFAP (Figure 4D, 4E, 4F and Figure 5D, 5E, 5F), but not with the neuronal marker NeuN (Figure 4A, 4B, 4C and Figure 5A, 5B, 5C) or the microglia marker OX-42 (Figure 4G, 4H, 4I and Figure 5G, 5H, 5I).

The expression of GLT-1 was significantly decreased in the ipsilateral spinal dorsal horn at 7(p < 0.01) and 14 days (p < 0.01) after PSNL (Figure 2C, 2D) compared with controls (Figure 2A). Semiquantitative analyses showed that the relative intensity of GLT-1 decreased by 42 ± 15.79% at 7 days and 32.3 ± 13.13% at 14 days following injury, respectively, when compared to the control group (Figure 2E). Similarly, the expression of GLAST was reduced by 54 ± 22.13% at 7 days and by 42 ± 16.34% at 14 days after nerve injury (Figure 3C, 3D, 3E). Decreases in the expression of GLT-1 and GLAST were also found significant in a side to side comparison of the contralateral versus ipsilateral sides of the spinal cord within the PNSL animals (Figure 2E and Figure 3E). However, the total amount of GLT-1 and GLAST one day after ligation did not change. The results from the above suggest that the decrease in GLT-1 and GLAST, which was induced by PNSL, mainly occurred in astrocytes on the injured side.

### Activation of microglia following PSNL

**Figure 6:**
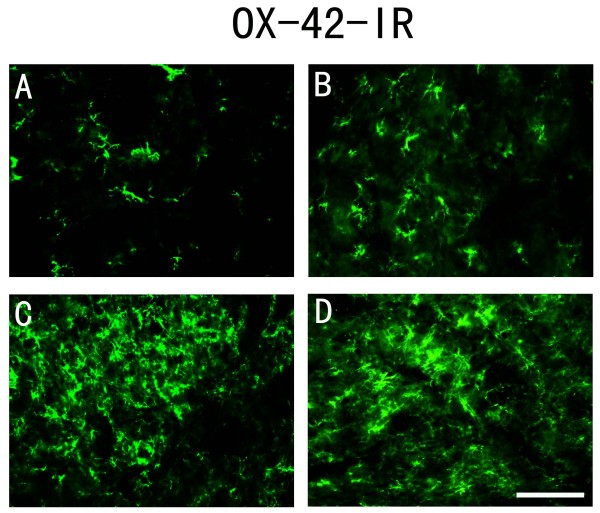
**Microglia were activated after PSNL**. A-D: OX-42-positve cells were increased in spinal dorsal horn 1 day (B), 7 days (C) and 14 days (D) after PSNL compared to the control group (A). Scale bar: (A-D) = 50 μm.

Microglia in the spinal horn also showed evidence of activation due to the PSNL. An increase in OX-42-positive cells were noted by day 1 post PSNL (Figure 6B) compared with the control group (Figure 6A). The increase in OX-42 appeared to reach a maximum on day 7 after nerve injury (Figure 6C) and maintained this elevated level through day 14 after PSNL (Figure 6D).

### Upregulation of GLT-1 and GLAST in microglia following PSNL

**Figure 7:**
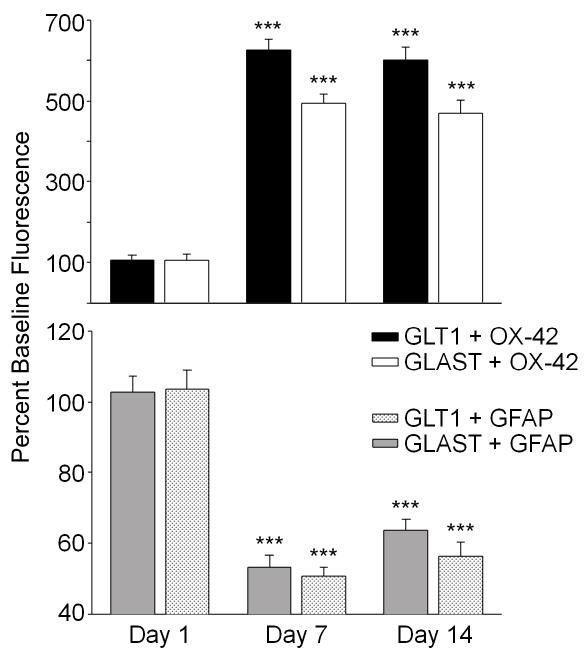
**The bar graphs show the intensity of double label fluorescence for GLT1-OX-42 (black bars), GLAST-OX-42 (open bars), GLT1-GFAP (gray bars), and GLAST-GFAP (cross-hatched bars)**. The intensity of double label of both glutamate transporters with GLT1 was significantly increased at days 7 and 14 following PSNL, whereas the intensity of double label for both transporters with GFAP was significantly decreased at these same time points. *** = p < 0.001.

**Figure 8:**
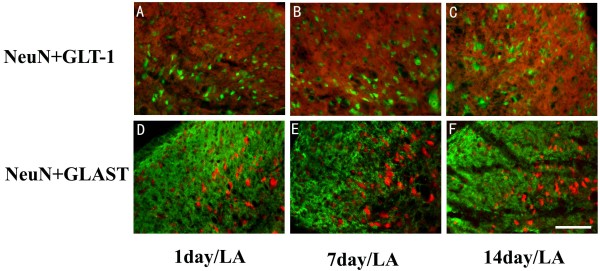
**Neither GLT-1 antibody-positive cells, nor GLAST antibody-positive cells co-localized with the NeuN-positive cells (Figure 7A)**. A-C: The NeuN-positive cells did not show any expression of GLT-1 1 day (A), 7 days (B) and 14 days (C) after PSNL. D-F: The NeuN-positive staining is not co-localized with the GLAST antibody-positive cells 1 day (D), 7 days (E) or14 days (F) after PSNL. Scale bar: (A-F) = 50 μm.

As noted above, there was no co-localization of GLT-1 or GLAST with OX-42 positive microglia in the absence of nerve injury. By day 7 post PSNL double staining showed that OX-42-positive cells (Figure 4O) showed a *de novo *up-regulation of GLT-1that persisted through day 14 post PSNL (Figure 5S). Likewise, GLAST-positive OX-42 positive cells were observed at both days 7 (Figure 4O) and 14 post PNSL (Figure 4S). Quantitative analysis of the intensity of co-labeling for the glutamate transporters with GFAP and OX-42 over time is shown in Figure 7. Significant decreases in double label intensity for both GLT-1 and GLAST with GFAP were found at days 7 and 14 following PSNL. The opposite was found for GLT-1 and GLAST co-localization with OX-42 with both showing increases at days 7 and 14 following PNSL. Finally, no co-localization of either GLT-1 or GLAST was found in NeuN positive cells (neurons) at any time point (Figure 8D, E).

## Discussion

The results of this study demonstrate plasticity not only in the expression levels but also in the cellular localization of the glutamate transporters GLT-1 and GLAST following partial sciatic nerve ligation. Staining of GLT-1 and GLAST was found in the spinal dorsal horn at baseline. GLT-1 and GLAST were not co-localized with the microglia marker OX-42 or the neuron marker NeuN at baseline, but rather only in cells positive for the astrocyte marker GFAP. This suggests that the expression of GLT-1 and GLAST under normal conditions in the spinal dorsal horn primarily originates in astrocytes. This conclusion is consistent with reports on the distribution of GLT-1 and GLAST in hippocampus and cerebellum where the transporters were found to be in astrocytes [18-21].

Nerve injury was found to result in a decrease in the total amount of GLT-1 and GLAST at 7 and 14 days after PSNL. The clear implication from the double-labeling study is that the synthesis of the transporters is down-regulated in astrocytes after PSNL. This result is consistent with other reports. For example, the total amount of GLT-1 decreased after facial nerve axotomy [22] and with spinal nerve injury [23]. Another line of investigation has shown that glutamate uptake is attenuated in spinal dorsal in rat after complete spinal nerve ligation [14]. Decreases in GLT-1 and GLAST transporters and an ensuing accumulation of glutamate in the synaptic cleft is consistent with neurophysiological studies of spinal neurons following nerve injury [24] wherein an excess of afterdischarges was noted as a cardinal feature. Conversely, gene transfer of GLT-1 into spinal cord attenuates inflammatory and neuropathic pain in rats [25]

Evidence of activation of spinal glia cells shown here by the increases in GFAP and OX-42 staining density is also consistent with previous reports [26-28]. An important implication from this work is that activated astrocytes evidently shift homeostatic functions upon exposure to nerve injury and part of this shift involves decreased regulation of synaptic functions evidenced by down-regulation of glutamate transporters. The mechanism by which astrocytes become activated remains an area of needed investigation. Potential signals may include central release of fractalkine or other growth factors [28]. Activation of spinal astrocytes appears to coincide with the initiation of re-growth of injured axons [29], which suggests that perhaps the shift in astrocyte function is to support the growth of central axons of primary afferent fibers that are in the process of re-generating across the lesion site. Evidence to support this possibility is that growth related antigens such as growth associated protein-43 and endogenous lectin RL-29 show peaks in the dorsal horn at the same time points where peaks in re-generating axons are found in injured nerve roots [30-33].

A novel finding from this work is the observation that PNSL-activated microglia initiate a new expression of GLT-1 and GLAST. In agreement with this observation, GLT-1 was observed as newly expressed in scattered microglia in an astrocyte-neuron culture [34]. This finding suggests that as astrocytes shift to support re-growth and the establishment of re-connectivity, that microglia assumes the role of regulating synaptic function.

Finally, no expression or change in expression of GLT-1 or GLAST was observed in neurons. The expression of the neuronal glutamate transporter EAAC1 was not explored here. Hence the potential for plasticity in this expression remains open to investigation.

## Conclusion

This paper provides evidence that not only the expression but also the cellular localization of the glutamate transporters changed following PSNL. Under baseline physiological conditions, the expression of GLT-1 and GLAST in the spinal dorsal horn is confined to astrocytes. After PSNL, spinal glial cells show evidence of activation, the expression of GLT-1 and GLAST are decreased, and the decreases occur in the astrocytes. Microglia activated following PSNL initiate a new expression of GLT-1 and GLAST.

## Methods

### Experimental animals

A total of 46 male Sprague-Dawley rats weighing 180–220 g were used. All experiments were conducted with the approval of the Institutional Animal Care and Use Committee at the M.D. Anderson Cancer Center and were in compliance with the National Institutes of Health Guidelines for Use and Care of Laboratory Animals. The minimum number of animals was used in each experiment, and in all cases every effort was made to minimize any pain or suffering in the subject animals.

### Surgical procedure

Rats were anesthetized with 50 mg/kg (i.p.) of pentobarbital and the left sciatic nerve was exposed at the high thigh level. Partial sciatic nerve ligation was performed as previously described [16,17]. Briefly, one-third to one-half of the left sciatic nerve was tightly ligated using 7-0 silk suture. Sham surgery in age matched animals consisted of exposing the left sciatic nerve but no ligation was made. The muscle layers were closed with 4-0 silk and the skin sealed with surgical clips.

### Behavioral testing

The rats were accommodated to the testing environment by placement within testing chambers for 15–20 min on the three separate days just prior to the pre-operative testing. Mechanical sensitivity was assessed using von Frey hairs as described previously[35]. Briefly, rats were placed under separate transparent Plexiglas chambers positioned on a wire mesh floor. Fifteen minutes were allowed for habituation. Each stimulus consisted of a 2–3 s application of the von Frey hair to the middle of the plantar surface of the foot with 5 min interval between stimuli. Brisk withdrawal or licking of the paw following the stimulus was considered a positive response. The experimenter who conducted the behavioral tests was blinded to all treatments.

### Immunohistochemistry

Rats were deeply anesthetized with urethane (1.5 g/kg, i.p.) at 1, 7 or 14 days following nerve injury, the chest opened, and then quickly perfused through the ascending aorta with warm heparinized saline, followed by 4% paraformaldehyde in 0.1 M phosphate buffer, pH 7.2–7.4, 4°C. The L4–L5 spinal segments were removed and post-fixed for 3 h in the same fixative, and then stored in 30% sucrose overnight. Transverse sections (25 μm) were cut by cryostat and processed for immunohistochemical staining as previously described [36]. Sections were blocked with 3% donkey serum in 0.3% Triton X-100 for 1 hour at room temperature then incubated overnight at 4°C with guinea pig anti-GLT-1 antibody (1:2000, Chemicon) or rabbit anti-GLAST antibody (1:250, Abcam). The sections were then incubated for 1 h at room temperature with FITC-conjugated secondary antibody (1:250, Chemicon) or Cy^3^-conjugated secondary antibody (1:500, Chemicon). For double immunofluorescence, the spinal sections were incubated with a mixture of guinea anti-GLT1 antibody or rabbit anti-GLAST antibody and mouse anti-neuronal nuclei (NeuN, neuronal marker, 1:500, Chemicon), mouse anti-glial fibrillary acidic protein (GFAP, Astrocyte marker, 1:500, Chemicon) or mouse anti-OX-42 (Microglia marker, 1:500, Chemicon) antibody overnight at 4°C. Afterwards the sections were incubated with a mixture of FITC- and Cy3-conjugated secondary antibodies for 1 h at a room temperature. The stained sections were then examined with a Nikon E600 (Nikon Instech Co, Japan) fluorescence microscope and images were captured with a CCD spot camera.

### Statistical analysis

The relative intensity of GLT-1-IR and GLAST-IR per section was measured in the spinal dorsal horn using a computerized image analysis system (NIS-Elements, BR 2.30). An optic threshold was set above background level firstly to identify positively stained structures. Relative intensity values used for comparison were calculated by multiplying the mean optic density in areas of interest and then subtracting out background staining (mean optic density * positive area – value of background). In each rat, four to six sections of the spinal cord at each time point were selected randomly. An average percentage of GLT-1-IR, GLT-1 + OX-42-IR, GLT-1 + GFAP-IR, GLAST-IR, GLAST + OX-42-IR, and GLAST + GFAP-IR relative to the control group, was obtained for each animal across different time points, and then the mean ± SE among the animals was determined. Four to eight rats were included for each group for quantification of the results. All measurements were performed by blinded evaluators. Data were compared with student's t-test or one-way ANOVA followed by Neuman-Keuls tests. P < 0.05 was considered significant.

## Abbreviations

GLT-1: glutamate-aspartate transporter; GLAST: excitatory amino acid carrier-1; PSNL: partial sciatic nerve ligation; NMDA: N-methyl-D-aspartate; AMPA: a-amino-3-hydroxy-5-methyl-4-isoxazolepropionic acid; GFAP: glial fibrillary acidic protein

## Competing interests

The authors declare that they have no competing interests.

## Authors' contributions

WJX carried out all the experiment and drafted the manuscript. HRW participated in the design of the study. PMD conceived of the study, and participated in the design and helped to draft the manuscript. All authors read and approved the final manuscript.

## References

[B1] Mayer ML, Westbrook GL (1987). The physiology of excitatory amino acids in the vertebrate central nervous system. Prog Neurobiol.

[B2] Danbolt NC (2001). Glutamate uptake. Prog Neurobiol.

[B3] Bennett GJ (2000). Update on the neurophysiology of pain transmission and modulation: focus on the NMDA-receptor. J Pain Symptom Manage.

[B4] Bennett GJ (2000). A neuroimmune interaction in painful peripheral neuropathy. Clin J Pain.

[B5] Rothstein JD, Dykes-Hoberg M, Pardo CA, Bristol LA, Jin L, Kuncl RW, Kanai Y, Hediger MA, Wang Y, Schielke JP, Welty DF (1996). Knockout of glutamate transporters reveals a major role for astroglial transport in excitotoxicity and clearance of glutamate. Neuron.

[B6] Wadiche JI, Arriza JL, Amara SG, Kavanaugh MP (1995). Kinetics of a human glutamate transporter. Neuron.

[B7] Gegelashvili G, Schousboe A (1997). High afinity glutamate transporters: Regulation of expression and activity. Mol Pharmacol.

[B8] Lehre KP, Danbolt NC (1998). The number of glutamate transporter subtype molecules at glutamatergic synapses: chemical and stereological quantification in young adult rat brain. J Neurosci.

[B9] Berger UV, Hediger MA (2000). Distribution of the glutamate transporters GLAST and GLT-1 in rat circumventricular organs, meninges, and dorsal root ganglia. J Comp Neurol.

[B10] Sonnewald U, Westergaard N, Schousboe A (1997). Glutamate transport and metabolism in astrocytes. GLIA.

[B11] Liaw WJ, Stephens RL, Binns BC, Chu Y, Sepkuty JP, Johns RA, Rothstein JD, Tao YX (2005). Spinal glutamate uptake is critical for maintaining normal sensory transmission in rat spinal cord. Pain.

[B12] Weng HR, Chen JH, Cata JP (2006). Inhibition of glutamate uptake in the spinal cord induces hyperalgesia and increased responses of spinal dorsal horn neurons to peripheral afferent stimulation. Neuroscience.

[B13] Sung B, Lim G, Mao J (2003). Altered expression and uptake activity of spinal glutamate transporters after nerve injury contribute to the pathogenesis of neuropathic pain in rats. J Neurosci.

[B14] Binns BC, Huang Y, Goettl VM, Hackshaw KV, Stephens RL (2005). Glutamate uptake is attenuated in spinal deep dorsal and ventral horn in the rat spinal nerve ligation model. Brain Res.

[B15] Weng HR, Aravindan N, Cata JP, Chen JH, Shaw AD, Dougherty PM (2005). Spinal glial glutamate transporters downregulate in rats with taxol-induced hyperalgesia. Neurosci Lett.

[B16] Seltzer Z, Dubner R, Shir Y (1990). A novel behavioral model of neuropathic pain disorders produced by partial sciatic nerve injury. Pain.

[B17] Dougherty PM, Garrison CJ, Carlton SM (1992). Differential influence of local anesthetic upon two models of experimentally-induced peripheral mononeuropathy in the rat. Brain Res.

[B18] Storck T, Schulte S, Hofmann K, Stoffel W (1992). Structure, expression, and functional analysis of a Na(+)-dependent glutamate/aspartate transporter from rat brain. Proc Natl Acad Sci USA.

[B19] Pines G, Danbolt NC, Bjoras M, Zhang Y, Bendahan A, Eide L, Koepsell H, Storm-Mathisen J, Seeberg E, Kanner BI (1992). Cloning and expression of a rat brain L-glutamate transporter. Nature.

[B20] Tanaka K (1993). Expression cloning of a rat glutamate transporter. Neurosci Res.

[B21] Gegelashvili G, Schousboe A (1997). High affinity glutamate transporters: regulation of expression and activity. Mol Pharmacol.

[B22] Lopez-Redondo F, Nakajima K, Honda S, Kohsaka S (2000). Glutamate transporter GLT-1 is highly expressed in activated microglia following facial nerve axotomy. Brain Res Mol Brain Res.

[B23] Tawfik VL, Regan MR, Haenggeli C, Lacroix-Fralish ML, Nutile-McMenemy N, Perez N, Rothstein JD, DeLeo JA (2008). Propentofylline-induced astrocyte modulation leads to alterations in glial glutamate promoter activation following spinal nerve transection. Neuroscience.

[B24] Palecek J, Paleckova V, Dougherty PM, Carlton SM, Willis WD (1992). Responses of spinothalamic tract cells to mechanical and thermal stimulation of the skin in rats with an experimental peripheral neuropathy. J Neurophysiol.

[B25] Maeda S, Kawamoto A, Yatani Y, Shirakawa H, Nakagawa T, Kaneko S (2008). Gene transfer of GLT-1, a glial glutamate transporter, into the spinal cord by recombinant adenovirus attenuates inflammatory and neuropathic pain in rats. Mol Pain.

[B26] Garrison CJ, Dougherty PM, Kajander KC, Carlton SM (1991). Staining of glial fibrillary acidic protein (GFAP) in lumbar spinal cord increases following a sciatic nerve constriction injury. Brain Res.

[B27] Ma W, Quirion R (2002). Partial sciatic nerve ligation induces increase in the phosphorylation of extracellular signal-regulated kinase (ERK) and c-Jun N-terminal kinase (JNK) in astrocytes in the lumbar spinal dorsal horn and the gracile nucleus. Pain.

[B28] Watkins LR, Milligan ED, Maier SF (2001). Glial activation: a driving force for pathological pain. Trends Neurosci.

[B29] Aldskogius H, Kozlova EN (1998). Central neuron-glial and glial-glial interactions following axon injury. Prog Neurobiol.

[B30] Curtis R, Tonra JR, Stark JL, Adryan KM, Park JS, Cliffer KD, Lindsay RM, DiStefano PS (1998). Neuronal injury increases retrograde axonal transport of the neurotrophins to spinal sensory neurons and motor neurons via multiple receptor mechanisms. Mol Cell Neurosci.

[B31] Cameron AA, Cliffer KD, Dougherty PM, Garrison CJ, Willis WD, Carlton SM (1997). Time course of degenerative and regenerative changes in the dorsal horn in a rat model of peripheral neuropathy. J Comp Neurol.

[B32] Cameron AA, Dougherty PM, Garrison CJ, Willis WD, Carlton SM (1993). The endogenous lectin RL-29 is transynaptically induced in dorsal horn neurons following peripheral neuropathy in the rat. Brain Res.

[B33] Cameron AA, Cliffer KD, Dougherty PM, Willis WD, Carlton SM (1991). Changes in lectin, GAP-43 and neuropeptide staining in the rat superficial dorsal horn following experimental peripheral neuropathy. Neurosci Lett.

[B34] Swanson RA, Liu J, Miller JW, Rothstein JD, Farrell K, Stein BA, Longuemare MC (1997). Neuronal regulation of glutamate transporter subtype expression in astrocytes. J Neurosci.

[B35] Cata JP, Weng HR, Dougherty PM (2008). The effects of thalidomide and minocycline on taxol-induced hyperalgesia in rats. Brain Res.

[B36] Xin WJ, Gong QJ, Xu JT, Yang HW, Zang Y, Zhang T, Li YY, Liu XG (2006). Role of phosphorylation of ERK in induction and maintenance of LTP of the C-fiber evoked field potentials in spinal dorsal horn. J Neurosci Res.

